# Diagnostic Accuracy of a Nocturia Single Question Scale as a Predictor of Severity of Lower Urinary Tract Symptoms in Men

**DOI:** 10.7759/cureus.77058

**Published:** 2025-01-07

**Authors:** Caroline S Silva, Carlos S Bellucci, Matheus A Alvaia, Eduardo Miranda, Jose Murillo B Netto, Cristiano Gomes, Ricardo B Tiraboschi, Jose Bessa

**Affiliations:** 1 Public Health, Universidade Estadual de Feira de Santana, Feira de Santana, BRA; 2 Urology, Uroclinica de Joinville, Joinville, BRA; 3 Urology, Universidade Estadual de Feira de Santana, Feira de Santana, BRA; 4 Urology, Unichristus, Fortaleza, BRA; 5 Urology, Federal University of Juiz de Fora, Juiz de Fora, BRA; 6 Urology, Hospital das Clinicas, Sao Paulo, BRA

**Keywords:** health services accessibility, lower urinary tract symptoms, nocturia, prostatic hyperplasia, validation study

## Abstract

Objective: This study evaluates the efficacy of the Nocturia Severity Quality Score (NSQS) as a simplified tool for assessing the severity of lower urinary tract symptoms (LUTS) in men, comparing it to the International Prostate Symptom Score (IPSS), the established standard.

Methods: We conducted a cross-sectional analysis on 697 men aged ≥40 from two urban urology clinics in Brazil. Participants completed both the IPSS and the NSQS, the latter consisting of a single question assessing nocturia frequency on a scale from 0 to 4. Diagnostic accuracy was evaluated using receiver operating characteristic (ROC) curve analysis to compare the NSQS against the IPSS.

Results: The NSQS effectively distinguished between moderate/severe and mild/asymptomatic LUTS, achieving an area under the ROC curve of 0.75 (95% CI: 0.72-0.79). NSQS thresholds of ≥2 and ≥3 episodes per night corresponded to increased likelihoods of moderate to severe LUTS, with significant diagnostic value despite varying sensitivities and specificities.

Conclusion: The NSQS provides a valid, efficient alternative to the IPSS for the initial assessment of LUTS severity in men.

## Introduction

Lower urinary tract symptoms (LUTS) encompass a range of problems with storage, voiding, and post-micturition symptoms, significantly affecting men worldwide [1]. Benign prostatic hyperplasia (BPH) is one of the most common urological diseases associated with progressive LUTS [2]. The impact of these symptoms extends beyond mere discomfort, often disturbing sleep, reducing quality of life, and indicating potential underlying pathologies [3].

In clinical practice, accurate assessment of LUTS is crucial for effective management and treatment, particularly in aging populations where prevalence increases [4]. The International Prostate Symptom Score (IPSS) is an eight-item questionnaire comprising seven symptom questions and one quality of life question, the recognized standard for evaluating LUTS severity [5-6]. It has been validated in many countries and enables the stratification of patients according to the severity of symptoms [7-9]. Despite its widespread validation, its length and complexity pose challenges, particularly in settings with limited time and resources [10,11]. Literacy and cultural differences can skew patients' comprehension of the questions, potentially misrepresenting symptom severity [10].

In Brazil, the diversity in educational backgrounds and constrained healthcare resources highlight the necessity for a more accessible diagnostic tool [12,13]. This need is particularly critical in primary care, where urologists may be scarce, and patient volume is high [14].

Simplified diagnostic tools such as single-question surveys have shown efficiency in various medical fields [15-18]. Single-question questionnaires provide a brief and easily administered tool for detecting diseases and have been recommended as a screening tool for different specific medical conditions. For LUTS, a simple yet reliable question focusing on nocturia, a symptom with a significant negative impact, could streamline the initial evaluation process and guide further diagnostic and therapeutic decisions.

Previously, the single-question nocturia score proved to be an accurate and convenient tool for managing BPH patients [19]. Our objective was to evaluate how accurately the single-question scale assessing the frequency of nocturia episodes (NSQS) can determine LUTS severity in a Brazilian male population. The rationale for using the NSQS is its simplicity, as it saves time for both patients and clinicians, reduces the burden on respondents, and is especially useful in busy clinical settings or populations with low literacy. By proposing a streamlined approach, we aimed to provide a practical alternative to IPSS that accommodates the constraints of diverse clinical environments and improves patient care efficacy. An initial version of this article was previously published on a preprint server [20].

## Materials and methods

Study population

We conducted a cross-sectional study between July and December 2019. The study participants were men aged ≥40. They were recruited from regular follow-up appointments at two urological clinics in different cities in Brazil. Subjects with active urinary tract infections or those who had experienced such an infection within the previous month were excluded.

All methods, definitions, and units follow the International Continence Society's standards to ensure rigor and reproducibility [1].

This observational study was approved by the Research Ethics Committee of the State University of Feira de Santana on May 9, 2017, under protocol no. 64704017.7.0000.0053, position statement 2.052.761. All participants provided written informed consent.

LUTS assessment

Nocturia was defined as any need to void during the main sleeping period, with each micturition event preceded and followed by sleep [1].

LUTS was assessed using the self-administered IPSS validated version. A research assistant clarified any uncertainties regarding the questionnaire content for patients with low literacy levels who experienced comprehension difficulties. The IPSS consists of seven questions addressing urinary symptoms: sensation of incomplete emptying, frequency, intermittency, urgency, weak stream, straining, and nocturia. Responses to the first six questions were scored on a scale of 0 (none), 1 (less than one in five), 2 (less than half the time), 3 (about half the time), 4 (more than half the time), and 5 (almost always). The last question, which evaluates nocturia frequency, was scored in six categories, from none to five or more times. These questions form a scale by summing the responses (0-5 for each response), and patients may be categorized as asymptomatic (0 points), mild symptoms (1-7 points), moderate symptoms (8-19 points), and severe symptoms (20-35 points).

The Nocturia Severity Quality Score (NSQS) was obtained from the last question of the IPSS that evaluates nocturia. It was scored in five ordered categories, from none to five or more times, and was administered independently to all patients (Appendix A).

Sample size calculation and diagnostic accuracy evaluation

Considering a minimal disease prevalence of 15% according to previous studies evaluating LUTS in the male population [21], a sample of at least 615 subjects would be necessary for 80% sensitivity and specificity, with an absolute precision of 5%.

We investigated the diagnostic properties of the NSQS (index test) for categorization of LUTS severity based on the IPSS score (reference standard). We recorded the time to complete each questionnaire to gauge respondents’ burden.

This article complies with the recommendations of the Standards for Reporting of Diagnostic Accuracy initiative as a study of accuracy [22].

Statistical analysis

Data are presented as absolute values, frequencies, medians, and interquartile ranges (IQR). The sensitivity, specificity, predictive values, and likelihood ratio of each NSQS, including 95% confidence intervals (CI), describe the diagnostic accuracy. Receiver operating characteristic (ROC) curves were generated to visualize and calculate the area under the curve (AUC) used to describe the diagnostic characteristics of the NSQS in diagnosing the severity of LUTS. Statistical analyses were performed using GraphPad Prism, version 8.4.0 (GraphPad Software, San Diego, United States).

## Results

Of the initial 763 patients enrolled, 697 were included in the final analysis. About 37 were excluded due to current or recent urinary tract infections, and 29 refused to participate. The median age of the participants was 60.0 (54.0-68.0) IQR years. The median IPSS was 9.0 (5.0-17.0) IQR, and the median NSQS was 2.0 (1.0-3.0) IQR.

Prevalence of LUTS

According to the IPSS, the severity of symptoms among the participants was classified as mild in 262 (37.6%), moderate in 279 (40.0%), and severe in 129 (18.6%) patients. Twenty-seven (3.8%) patients were asymptomatic.

**Figure 1:**
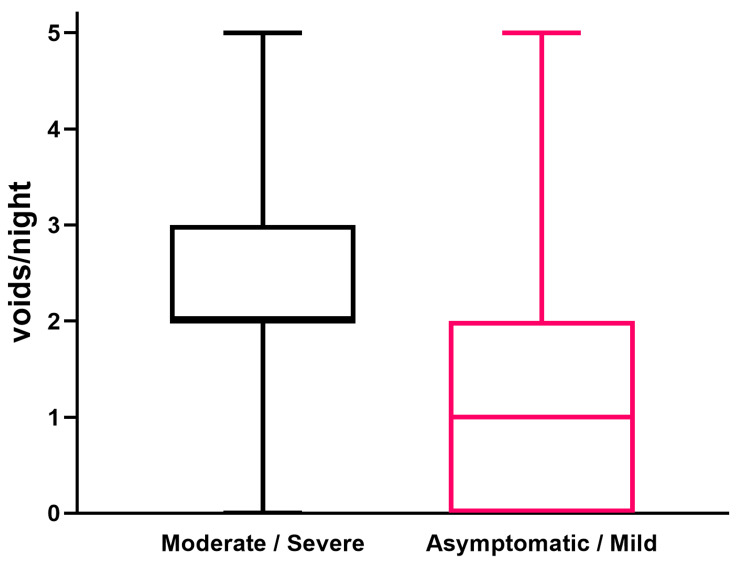
Box plot of nocturia episodes in subjects with moderate/severe versus asymptomatic/mild LUTS LUTS: lower urinary tract symptoms

The NSQS was zero in 118 patients (17%), one in 141 (20%), two in 197 (28%), three in 144 (21%), and four or more in 95 (13%). The NSQS was significantly higher in subjects with moderate/severe LUTS than in asymptomatic and mild symptoms, respectively, with 2.0 (2.0-3.0) IQR and 1.0 (0.0-2.0) IQR (p < 0.001). These data are detailed in Figure 1.

NSQS diagnostic properties

**Figure 2:**
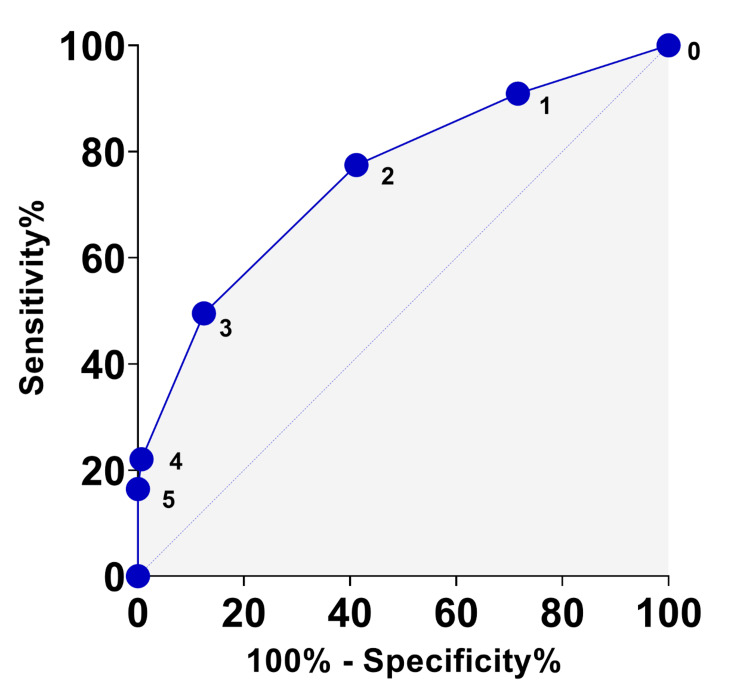
ROC curves for NSQS in distinguishing between patients with absent/mild (IPSS < 8) and moderate/severe (IPSS ≥ 8) LUTS ROC curves: receiver operating characteristic curves; NSQS: nocturia single question scale; IPSS: international prostate symptom score; LUTS: lower urinary tract symptoms

The NSQS demonstrated good overall accuracy in distinguishing more severe LUTS, as indicated by an area under the ROC curve of 0.75 (95% CI 0.72-0.79). Numbers 0-5 refer to episodes of nocturia (Figure 2).

Table 1 presents detailed analyses of sensitivity, specificity, and likelihood ratios for each NSQS threshold.

**Table 1:** Diagnostic parameters of NSQS thresholds for discriminating between patients with mild (IPSS < 8) and moderate/severe (IPSS ≥ 8) LUTS NSQS: nocturia single question scale; IPSS: international prostate symptom score; CI: confidence intervals; LR+: positive likelihood ratio; LR-: negative likelihood ratio; LUTS: lower urinary tract symptoms

Number of nocturia episodes				
NSQS	1	2	3	4
Sensitivity (95% CI)	90.9% (87.7-93.3)	77.4% (73.1-81.2)	49.5% (44.6-54.5)	22.0% (18.1-26.4)
Specificity (95% CI)	28.4% (23.5-33.8)	58.4% (52.7-64.0)	87.2% (82.8-90.8)	98.2% (96.0-99.4)
LR+	1.27	1.86	3.87	12.75
LR-	0.32	0.39	0.58	0.49
Youden index	119.3	135.8	136.7	120.2

The median time required to complete the NSQS was significantly shorter compared to the IPSS, with median times of 0.28 (0.12-0.45) IQR minutes and 2.7 (2.3-3.5) IQR minutes, respectively (p < 0.001).

## Discussion

This study demonstrates that the NSQS is an effective tool for assessing the severity of LUTS in adult men. Its overall accuracy (AUC of 0.75) supports its use in primary care. The NSQS, particularly at cut-offs of 2 and 3 nocturia episodes, demonstrated robust diagnostic value, offering a viable alternative to the more cumbersome IPSS.

NSQS ≥ 2 is particularly relevant as it is the standard number of voids considered in the definition of nocturia, with most authors recognizing two voids per night as the clinical threshold that notably affects quality of life [23-25]. Conversely, NSQS ≥ 3 demonstrates slightly higher overall accuracy and superior specificity, making it highly effective in predicting moderate to severe LUTS despite lower sensitivity, a feature precious in primary care settings where resources and specialist availability are limited. A previous study [20] has also investigated the role of a single nocturia question in the evaluation of 162 African men aged over 50 and revealed NSQS ≥ 3 as the most effective threshold, showing higher sensitivity (87.0%) and specificity (91.0%) for distinguishing severity of LUTS, in contrast to our findings, which may reflect regional educational and cultural differences affecting patient responses.

Other efforts have been applied to create simplified methods capable of evaluating LUTS in adult men, such as the Urgency, Weak stream, Incomplete emptying, and Nocturia (UWIN) questionnaire, recently validated in Brazilian Portuguese by our group [26-27]. UWIN provides comparable results to the IPSS, using a more straightforward format and taking less time to complete. However, it comprises a four-question questionnaire with scores ranging from 0 to 3, adding to a composite score of 0 to 12. The UWIN, although simpler than IPSS, might still add considerable effort from respondents and physicians. Other abbreviated Patient-Reported Outcome Assessments (PROs) have been proposed to decrease the burden of IPSS on respondents and clinicians, such as the Quick Prostate Test (QPT) and Frequency, Leakage, Overnight voiding, and Weak stream (FLOW) [28,29]. These instruments demonstrated valid equivalence to the IPSS and some advantages, including efficiency and ease of application.

The methodology employed in this study leveraged a cross-sectional design and utilized a single-question NSQS. It contrasts with the more complex IPSS, traditionally recognized for its thoroughness but criticized for its length and difficulty, especially in diverse educational backgrounds [10,11]. Our approach aimed to simplify the diagnostic process without compromising the quality of assessment, particularly in settings constrained by time and resources [12,13].

The predominantly urban, well-educated male demographic in this study might limit the generalizability of our findings to broader populations. This aspect is particularly relevant as lower educational levels have been shown to impact the comprehension and effectiveness of self-administered questionnaires like the IPSS [10,11,30].

Simplified tools such as the NSQS can significantly enhance diagnostic efficiency, improve patient throughput, and increase satisfaction in healthcare settings with limited resources. These tools reduce the burden on patients and healthcare providers, facilitating quicker clinical decision-making [27-29].

While our findings are promising, they must be interpreted with caution due to some limitations. This study was a non-randomized cohort of Portuguese-speaking Brazilian men in two tertiary urological centers in an urban region. The demographic profile of our respondents, primarily urban males with a high education level, potentially influences our findings' generalizability. Additionally, the study relied on self-reported data for assessing symptoms, which may introduce recall bias and subjectivity, potentially affecting the accuracy of the findings. Future research should aim to validate the NSQS across more diverse populations to enhance its applicability and reliability in different clinical and cultural contexts.

## Conclusions

This study establishes the NSQS as a practical, efficient, and reliable tool for predicting the severity of LUTS in men. The NSQS balances simplicity with good diagnostic accuracy. Our findings demonstrate that patients with one or no voids per night have a low probability of severe LUTS, while those reporting three or more voids are likely to experience moderate to severe symptoms. This clinical tool can optimize clinical care and potentially reduce clinician burden. It is suitably used in resource-limited settings and among populations with varying literacy levels.

## Figures and Tables

**Figure 3:**
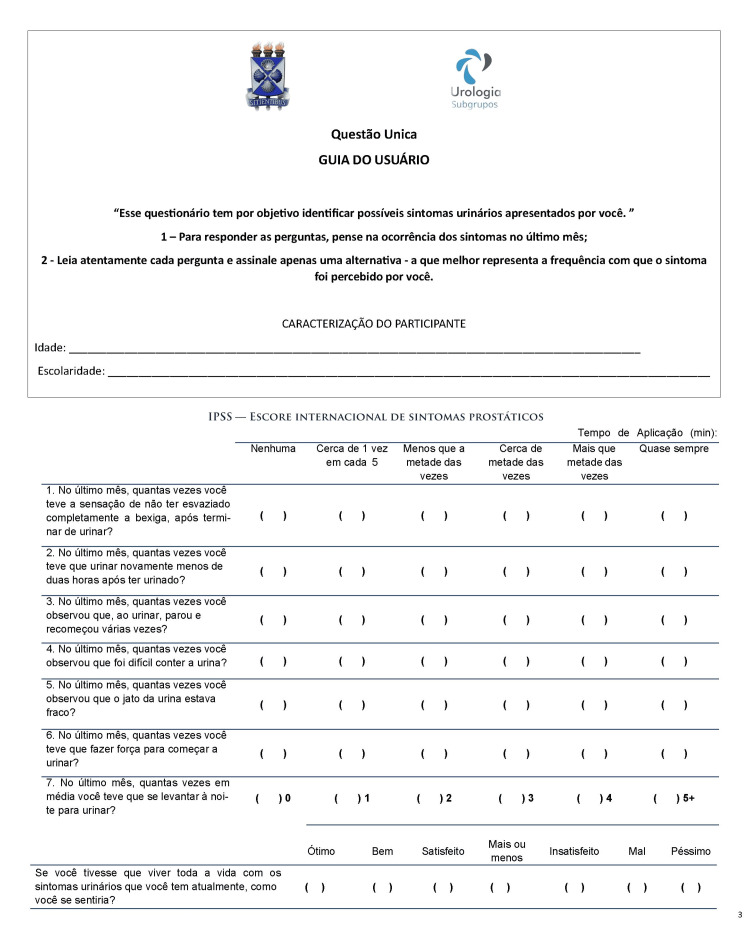
Questionnaire (Portuguese version)

**Figure 4:**
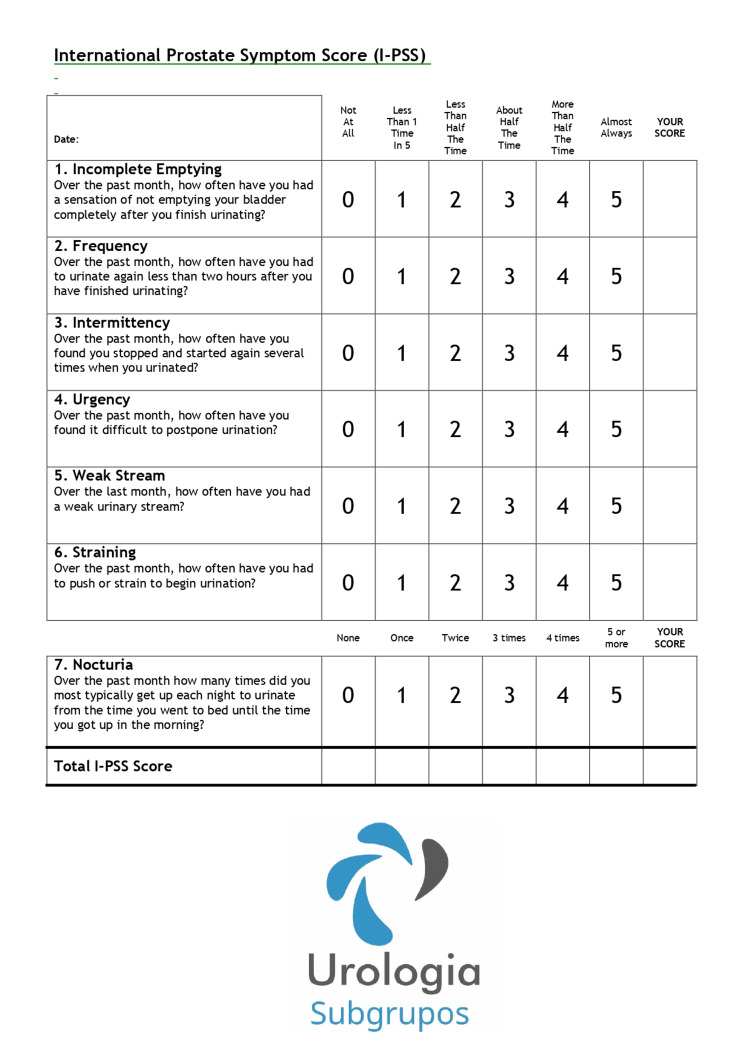
Questionnaire (English version)

## Appendices

Appendix A 

## References

[1] Ancona CD, Hamid R, Schizas A (2019). The International Continence Society (ICS) report on the terminology for adult male lower urinary tract and pelvic floor symptoms and dysfunction. Neurourol Urodyn.

[2] Speakman M, Kirby R, Doyle S, Ioannou C (2015). Burden of male lower urinary tract symptoms (LUTS) suggestive of benign prostatic hyperplasia (BPH) - focus on the UK. BJU Int.

[3] Soler R, Averbeck MA, Koyama MA, Gomes CM (2019). Impact of LUTS on treatment-related behaviors and quality of life: a population-based study in Brazil. Neurourol Urodyn.

[4] Haltbakk J, Hanestad BR, Hunskaar S (2005). How important are mens lower urinary tract symptoms (LUTS) and their impact on the quality of life (QOL)?. Qual Life Res.

[5] Ziadeh T, Mjaess G, El Helou J (2022). Impact on quality of life in multiple sclerosis patients: which urinary symptoms are to blame?. Prog Urol.

[6] Sandhu JS, Bixler BR, Dahm P, Goueli R, Kirkby E, Stoffel JT, Wilt TJ (2024). Management of lower urinary tract symptoms attributed to benign prostatic hyperplasia (BPH): AUA guideline amendment. J Urol.

[7] Jindal T, Sinha RK, Mukherjee S, Mandal SN, Karmakar D (2014). Misinterpretation of the international prostate symptom score questionnaire by Indian patients. Indian J Urol.

[8] Badía X, García-Losa M, Dal-Ré R, Carballido J, Serra M (1998). Validation of a harmonized Spanish version of the IPSS: evidence of equivalence with the original American scale. Urology.

[9] Barry MJ, Fowler FJ, O’Leary MP (1992). The American Urological Association symptom index for benign prostatic hyperplasia. J Urol.

[10] Ogwuche EI, Dakum NK, Amu CO, Dung ED, Udeh E, Ramyil VM (2013). Problems with administration of international prostate symptom score in a developing community. Ann Afr Med.

[11] Johnson T V., Abbasi A, Ehrlich SS (2008). Patient misunderstanding of the individual questions of the American Urological Association symptom score. J Urol.

[12] Machado CV, Silva GA (2019). Political struggles for a universal health system in Brazil: successes and limits in the reduction of inequalities. Global Health.

[13] Dantas MNP, Souza DLB de, Souza AMG de, Aiquoc KM, Souza TA de, Barbosa IR (2020). Factors associated with poor access to health services in Brazil (Article in Portuguese, English). Rev Bras Epidemiol.

[14] Juliani C, MacPhee M, Spiri W (2017). Brazilian specialists’ perspectives on the patient referral process. Healthcare (Basel).

[15] Aji BM, Larner AJ (2017). Screening for dementia: single yes/no question or Likert scale?. Clin Med (Lond).

[16] Maguire PA, Reay RE, Beverley R (2016). Correlates of a single-item self- rated mental health question in people with schizophrenia. Australas Psychiatry.

[17] Chu AH, Ng SH, Koh D, Müller-Riemenschneider F (2018). Domain-specific adult sedentary behaviour questionnaire (ASBQ) and the GPAQ single-item question: a reliability and validity study in an Asian population. Int J Environ Res Public Health.

[18] Min KD, Chun H, Kim IH, Cho SI (2018). Validating a single-question depression measure among older adults. Int Psychogeriatr.

[19] Kajimotu T, Bowa K (2018). Accuracy of a "Single Question Nocturia Score" compared to the "International Prostate Symptoms Score" in the evaluation of lower urinary tract symptoms in benign prostatic hyperplasia: a study performed at Ndola Teaching Hospital, Ndola, Zambia. PLoS One.

[20] Silva CS, Bellucci CHS, de Paula Miranda E (2020). Diagnostic accuracy of a nocturia single question scale as a predictor of severity of lower urinary tract symptoms in men [PREPRINT]. medRxiv.

[21] Soler R, Gomes CM, Averbeck MA, Koyama M (2018). The prevalence of lower urinary tract symptoms (LUTS) in Brazil: results from the epidemiology of LUTS (Brazil LUTS) study. Neurourol Urodyn.

[22] Bossuyt PM, Reitsma JB (2003). The STARD initiative. Lancet.

[23] Nakagawa H, Niu K, Hozawa A (2010). Impact of nocturia on bone fracture and mortality in older individuals: a Japanese longitudinal cohort study. J Urol.

[24] Tikkinen KA, Johnson TM 2nd, Tammela TL, Sintonen H, Haukka J, Huhtala H, Auvinen A (2010). Nocturia frequency, bother, and quality of life: how often is too often? A population-based study in Finland. Eur Urol.

[25] Åkerla J, Pesonen JS, Pöyhönen A (2022). Lower urinary tract symptoms and mortality among Finnish men: the roles of symptom severity and bother. J Urol.

[26] Eid K, Krughoff K, Stoimenova D, Smith D, Phillips J, O'Donnell C, Barqawi A (2014). Validation of the Urgency, Weak stream, Incomplete emptying, and Nocturia (UWIN) score compared with the American Urological Association Symptoms Score in assessing lower urinary tract symptoms in the clinical setting. Urology.

[27] Silva CS, Freitas KS, R Ribeiro AP, Gomes CM, Bessa Junior J (2020). Transcultural adaptation and validation of the questionnaire "Urgency, Weak stream, Incomplete emptying and Nocturia (UWIN)" for the Brazilian Portuguese. PeerJ.

[28] Moses KA, Heslop D, Griffith DM (2017). Development and initial testing of the FLOW instrument, a novel assessment of lower urinary tract symptoms in men. J Urol.

[29] Albino G, Niro CM, Muscarella C (2014). Quick Prostate Test (QPT): motion for a tool for the active contribution of the general pratictioner to the diagnosis and follow up of benign prostatic hyperplasia. Arch Ital Urol Androl.

[30] Martin SA, Haren MT, Marshall VR, Lange K, Wittert GA (2011). Prevalence and factors associated with uncomplicated storage and voiding lower urinary tract symptoms in community-dwelling Australian men. World J Urol.

